# Does work-personal life interference predict turnover among male and female managers, and do depressive symptoms mediate the association? A longitudinal study based on a Swedish cohort

**DOI:** 10.1186/s12889-018-5736-7

**Published:** 2018-07-04

**Authors:** Anna Nyberg, Paraskevi Peristera, Claudia Bernhard-Oettel, Constanze Leineweber

**Affiliations:** 1 Stress Research Institute, Stockholm University, SE-106 91 Stockholm, Sweden; 2 Department of Psychology, Stockholm University, SE-106 91 Stockholm, Sweden

**Keywords:** Work-personal life interference, Actual turnover, Depressive symptoms, Mediation, Autoregressive cross-lagged model, Multilevel structural equation model, Longitudinal

## Abstract

**Background:**

In the present study we used a longitudinal design to examine if work-personal life interference predicted managerial turnover, if depressive symptoms mediated the association, and if the relationships differed by gender.

**Methods:**

Data were drawn from four waves (2010, 2012, 2014 and 2016) of the Swedish Longitudinal Occupational Survey of Health (SLOSH), a cohort of the Swedish working population. Participants who in any wave reported to have a managerial or other leading position were included (*n* = 717 men and 741 women). Autoregressive longitudinal mediation models within a multilevel structural equation modelling (MSEM) framework, in which repeated measures (level 1) were nested within individuals (level 2), were fitted to data. First, bivariate autoregressive and cross-lagged paths between the variables were fitted in gender stratified models. Secondly, a full gender stratified mediation model was built to estimate if the association between work-personal life interference and turnover was mediated through depressive symptoms. Gender differences in cross-lagged paths were estimated with multiple-group analysis. All analyses were adjusted for age, education, labour market sector, civil status and children living at home, and conducted in MPLUS 7.

**Results:**

In both genders there were significant paths between work-personal life interference and turnover. Depressive symptoms were, however, not found to mediate in the relationship between work-personal life interference and turnover. The models differed significantly between genders.

**Conclusions:**

Establishing organisational prerequisites for good work-personal life balance among managers may be a means to retain both male and female managerial talent.

## Background

### Turnover among managers

Recruiting employees with leadership skills to managerial positions in organisations and retaining them within managerial careers has been discussed as particularly difficult in Northern Europe, [1] but acknowledged in large parts of the western world [2]. Stress and poor work-personal life balance are referred to as key reasons for why employees refrain from entering managerial careers [1]. Women moreover often express a lower interest in managerial careers [3] and it is sometimes reported that managerial turnover is higher among women [4]. However, relatively few studies have to date investigated antecedents of actual turnover among supervisors or managers [5–10]. In order to attain a greater gender equality at the top of work organisations, [11] a better understanding of turnover processes among both female and male managers appears crucial [12]. In a few studies of the Swedish public sector, poor psychosocial working conditions have been identified as antecedents to managerial turnover [5, 7, 13]. A lack of job control was found to be associated with subsequent internal turnover among male and female managers in Swedish healthcare organisations [7]. Also, interpersonal problems were associated with turnover intentions and to some extent actual turnover among managers in Swedish municipalities [5]. Knudsen et al. furthermore found high job demands to be associated with turnover intentions among leaders of addiction treatment organizations in the U.S., and emotional exhaustion to partially mediate this association [9]. In the present study we use a Swedish national survey to investigate if work-personal life interference predicts turnover among managers, if this relationship is mediated through depressive symptoms, and if the associations differ between genders.

### Work-personal life interference and managerial turnover

Although the interface between work and personal life has been recognized as a key issue for managers, it has seldom been empirically investigated in relation to turnover [10, 14]. A few studies have focused on work-family conflict as an antecedent to turnover intentions, [15, 16] but turnover intentions and actual turnover have been found to measure different phenomena [5]. In the present study we also use work-personal life interference, a concept expanded from work family conflict to include also other roles in personal life than those associated with family, as the predictor [17]. This includes for example neglecting own personal needs and not being able to maintain the kind of personal life one would like to have because of work. Swedish managers have been found to report higher levels of work-personal life interference than non-managers, [18] and work-personal life interference has previously been linked to turnover among employees [19]. Our first hypothesis is consequently that difficulties to combine a managerial position with a rich or demanding personal life could be a reason for managers to leave their position.

### The mediating role of depressive symptoms

The mediating role of depressive symptoms in the association between work-personal life interference and managerial turnover has, to our knowledge, not previously been investigated. Difficulties to combine a professional career with a rich or demanding personal or family life have been found linked to increased risk of stress, depression and sickness absence among employees in general [20–24]. Employee mental ill-health is, however, relatively poorly investigated as antecedents of turnover [6, 25]. Experienced strain has been found to be prospectively associated with voluntary turnover among Dutch truck-drivers [26] and experienced psychological strain and burnout associated with turnover among employees in a nationwide US engineering firm [27]. Furthermore, as stated above, emotional exhaustion has been found to partially mediate the association between job demands and turnover intentions among leaders in a north American study [9]. However, it is reasonable to assume that work-personal interference in terms of neglecting personal needs and not being able to do the things one would like to do due to work may lead not only to experienced stress and lack of recuperation, but over time also to depressive symptoms, such as feeling low in energy, feeling sad, and feeling a lack of interest in things [23]. Such symptoms may in turn lead to either that managers make the decision to quit their managerial careers in order to restore work-life balance and improve mental health, or that they are dismissed from their managerial position because the mental ill-health interferes with the fulfilment of their assignment. Thus, our second hypothesis is that depressive symptoms partially mediate the association between work-personal interference and managers’ actual turnover.

### Gender differences

Women do considerably longer hours of unpaid household work than men also in a relatively gender equal country like Sweden, [28] which appears to hold true also when comparing male and female Swedish managers [18]. Swedish female managers have also reported higher levels of work-personal interference than male managers [18]. Previous studies have found work-family conflict/work personal life interference to be a significantly stronger predictor of depressive symptoms among women than men [24, 29]. Our third hypothesis is consequently that the associations between work-personal life interference, depressive symptoms and turnover differ by gender and that depressive symptoms play a more important intermediate role for female than male managers.

### Reverse associations

Individuals higher in depressive symptoms may due to their mental state interpret situations and events differently than individuals lower in depressive symptoms and interact differently with these [30]. It is also possible that leaving a managerial position influences depressive symptoms and experiences of to what extent work interferes with personal life. Because of this also the reverse associations between the variables are estimated in the present study.

### Aim of the present study

In the present study, using longitudinal data from a cohort of the Swedish working population, we investigated work-personal life interference as an antecedent of female and male managers’ turnover, and depressive symptoms as a possible mediator in this relationship. The specific research objectives were to examine whether:

1. Work-personal life interference predict turnover in the consecutive two years among male and female managers.
2. Depressive symptoms mediate the association between work-personal life interference and turnover.
3. The associations between work-personal life interference, depressive symptoms and turnover differ by gender.

## Methods

### Study sample

Data were derived from the Swedish Longitudinal Occupational Survey of Health (SLOSH), which is based on an approximately nationally representative sample of the Swedish working population. SLOSH started in 2006 as a follow-up to the 2003 Swedish Work Environment Surveys (SWES). Respondents from SWES 2005 were added in the 2008 SLOSH wave, and participants from SWES 2007, 2009 and 2011 have been invited to participate in later SLOSH follow-ups. These are conducted every second year and the participants are followed by means of postal self-completion questionnaires in two versions: one for those currently in paid work at least 30% of full time and one for those working less or not at all. A more detailed description of the SLOSH study can be found elsewhere [31]. The present study includes four SLOSH waves from 2010 to 2016. Earlier waves could not be used because work-personal interference was not measured until 2010. In SLOSH 2010 a total of 11,525 participants responded to the questionnaire. The response rate varied between 51 and 57% over the follow-ups in 2012, 2014, and 2016. In the present study we included participants who responded to the questionnaire for those who were in paid work at least 30% in all four waves and in at least one of them reported that they had a managerial position. After exclusion of the self-employed the final study sample comprised 1458 participants, 741 women and 717 men. The Regional Research Ethics Board in Stockholm approved the present study (2016/232–31/5).

### Study variables

All study variables are measured in 2010, 2012, 2014 and 2016.

#### Outcome variable

Turnover was measured with the question: “Have you changed your job position over the past 2 years?” The response alternatives were “yes, to a higher position”, “no, I have not changed position”, and “yes, to a lower position”. Respondents who reported that they had changed to a lower position over the past 2 years were categorized into having left their managerial position (turnover), whereas those who indicated that they had stayed on the same level or had got a higher position made up the reference category.

#### Predictor variable

Interference between work and personal life was measured with an adapted version of a questionnaire developed by Fisher, [17] a questionnaire designed to measure directions and domains of work personal life interference and enhancement. For the multilevel structural equation models four statements (“I come home too tired to do things I would like to do”, “My personal life suffers because of my work”, “My job makes it difficult to maintain the kind of personal life I would like”, and “I often neglect my personal needs because of the demands of my work”) were used as indicators for the latent variable work-personal life interference. The indicators were analyzed as continuous variables and the five response alternatives ranged from 1, “not at all” to 5, “almost all the time” [17, 32]. To provide the reader with information on the level of work-personal life interference in the study sample (see Table 1) a mean index score was calculated by summing up the values of each indicator and dividing by the number of indicators (Cronbach’s alpha 0.89).

**Table 1:** Distribution of study variables for men (*n* = 717) and women (*n* = 741) separately in SLOSH 2010

	Men		Women		Gender diff
	*n/means*	*%/std dev*	*n/means*	*%/std dev*	*level of sign*
Turnover					n.s.
no	685	97.0	713	98.1	
yes	21	3.0	14	1.9	
Work-personal life interference (index mean/std. dev, range 1–5)	2.67	0.91	2.87	0.94	<.001
Depressive symptoms (index mean/std. dev, range 1–5)	1.74	0.80	1.92	0.86	<.001
Age					<.05
1 < 34 years	54	7.5	46	6.2	
2 35–44 years	198	27.6	231	31.2	
3 45–54 years	290	40.4	293	39.5	
4 55–64 years	160	22.3	168	22.7	
5 > 64 years	15	2.1	3	0.4	
Education					<.001
1 ≤ 9 years	38	5.3	21	2.8	
2 ≤ 12 years	297	41.4	201	27.1	
3 University < 3 years	97	13.5	38	5.1	
4 University ≥3 years	262	37.1	461	62.2	
5 Research education	23	2.6	20	2.7	
Labour market sector					<.001
0 Private	416	68.1	230	34.9	
1 Public	195	31.9	429	65.1	
Marital status					<.01
0 Married/co-habiting	603	85.5	593	80.7	
1 Not married/co-habiting	102	14.5	142	19.3	
Children living at home					n.s.
0 No	258	36.5	298	40.5	
1 Yes	448	63.5	437	59.5	

#### Mediating variable

Depressive symptoms were measured with the Symptom Checklist-Core Depression Scale (SCL-CD_6_), a six-item subscale from the Symptom Checklist-90 [33]. The question “How much during the last week have you been troubled by…” was followed by six different core depressive symptoms (feeling blue/sad, feeling no interest in things, feeling low in energy, feeling that everything is an effort, worrying too much, and blaming yourself for various things). The five response alternatives reached from 1, “not at all” to 5, “very much”. For the multilevel structural equation models the six core symptoms were used as indicators of the latent variable depressive symptoms and analyzed as continuous variables. Information on the level of depressive symptoms in the study sample is given as a mean index score in Table 1 (Cronbach’s alpha 0.91).

#### Covariates

Age was adjusted for in the groups < 34 years, 35–44, 45–54, 55–64, and > 64 years old. Labour market sector was adjusted for in the groups private and public. Educational background was adjusted for in the categories ≤9 years of education, ≤ 12 years of education, < 3 years of university education, ≥ 3 years of university education, and research education. Marital status was adjusted for using the categories married/co-habiting or not, and having children living at home as a dichotomous variable with the response alternatives “yes” and “no”.

### Analytic strategy

#### Descriptive statistics

Descriptive statistics of the study population in 2010 were computed separately for men and women. Gender differences in the distribution of study variables were estimated with Pearson’s chi-squared test or one-way analysis of variance (ANOVA) in SPSS version 24.

#### Autoregressive cross-lagged mediation models within a multilevel structural equation modelling (MSEM) framework

Autoregressive cross-lagged mediation models within a multilevel structural equation modelling (MSEM) framework were fitted to our data [34, 35]. The autoregressive cross-lagged models allow simultaneously addressing the reciprocal temporal relationships between work-personal life interference (WPI, exposure variable), depressive symptoms (DEP, putative mediator), and turnover (TO, outcome). Since multiple measurement points (level 1) are nested within individuals (level 2), a two-level SEM approach that allows partitioning between- and within-person effects was implemented so as to account for two inherent types of heterogeneity, within person across time and between person [36–38]. Work-personal life interference and depressive symptoms were fitted as latent variables with four and six indicators. The fit of the measurement model was tested as well as measurement invariance in the latent variables work-personal life interference and depressive symptoms over time. First, a simultaneous equation model that allows for autoregressive and cross-lagged effects between work-personal life interference and turnover at each time point was estimated in a gender stratified model. Work-personal life interference was measured at the first time point (t-1, years 2010, 2012, and 2014) and turnover the subsequent time point (t, years 2012, 2014 or 2016). The cross-lagged paths estimated the effect of one variable on the other with a two-year time lag. Each path in the models was adjusted for age, education, labour market sector, civil status, and children living at home. Indicators of the latent variables work-personal interference and depressive symptoms were allowed to correlate between waves. To estimate if the cross-lagged paths differed between genders we conducted multiple-group analyses testing differences in each hypothesised and reverse association separately. We created two groups based on gender and compared a model in which the paths were allowed to vary freely with a model in which the paths were constrained to be equal between men and women. The likelihood ratio test was used for comparing restricted and not restricted models. A significant change in chi square (df) between the not restricted model and the restricted one indicates a poorer fit for the restricted model.

In a second step, following the same time structure as above, we examined the bivariate multilevel structural cross-lagged relationships between work-personal life interference (predictor variable) and depressive symptoms (putative mediator) as well as between depressive symptoms and turnover (outcome). The models were stratified by gender and adjusted for the same set of covariates as above. If there are significant paths between the predictor and the mediator and between the mediator and the outcome, a mediation model under the MSEM framework can be fitted. The third step was to apply such a model to our data. A gender stratified longitudinal mediation model within a MSEM framework, in which work-personal life interference was measured at t-2 (in the years 2010 or 2012) depressive symptoms at t-1 (in the years 2012 or 2014) and turnover at t (in the years 2014 or 2016), was fitted. Autoregressive effects as well as cross-lagged paths were estimated between work-personal life interference and depressive symptoms and between depressive symptoms and turnover. The model was adjusted for the same set of covariates as in the bivariate models. The direct effect (the part of the exposure effect which was not mediated through depressive symptoms) as well as the indirect effect (the part of the exposure effect which was mediated through depressive symptoms) between work-personal life interference and turnover were estimated.

The multilevel SEM models were built in MPLUS 7. All variables were treated as time-varying variables. Standardised estimates were reported for the final models. The fit statistics chi-square (df), the root mean square error of approximation (RMSEA), the comparative fit index (CFI), the Tucker-Lewis index (TLI), and the standardized root mean square residual (SRMR) were considered. Model fit is assumed to be acceptable when RMSEA ≤0.08, TLI ≥ 0.90, CFI ≥ 0.90, and SRMR ≤0.08 [39].

## Results

### Descriptive statistics

The distribution of the study variables measured in 2010 are presented in means/standard deviations or n/percentages separately for men and women in Table 1. The level of significance for gender differences is shown in the last column. Over the study period 191 managers reported having a lower position (indicating turnover) today compared with two years earlier. In 2010 this corresponded to 3% of the men and 1.9% of the women, in 2012 3.5% of the men and 2.6% of the women, in 2012 3.1% of the men and 3.6% of the women, and finally in 2016 3.9% of the men and 4.7% of the women. The level of work-personal interference (range 1–5) measured as index mean (standard deviation) was for men in 2012 2.54 (0.87), in 2014 2.62 (0.92), and in 2016 2.61 (0.94). Corresponding numbers for women were in 2012 2.71 (0.96), 2014 2.78 (1.00), and 2016 2.72 (1.00). The levels of depressive symptoms (range 1–5) measured as index mean (standard deviation) were among men in 2012 1.66 (0.75), 2014 1.72 (0.72), and 2016 1.68 (0.75). The corresponding numbers among women were in 2012 1.81 (0.84), 2014 1.88 (0.83), and 2016 1.82 (0.79). A higher percentage of female than male managers worked in the public sector and were unmarried, and the age and educational level also differed between genders. Furthermore, female managers reported higher levels of work-personal interference and depressive symptoms than male managers throughout the study period.

### Bivariate multilevel cross-lagged structural equation models between work-personal life interference and turnover for men and women

**Fig. 1:**
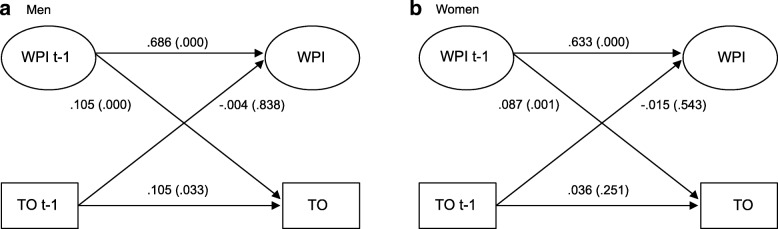
Lags and cross-lags in multilevel structural equation models for paths between work-personal life interference (WPI) and turnover (TO) at time t-1 (ranging from year 2010 to 2014) and t (years 2012 to 2016) among **a** men, *n* = 717 and **b** women, *n* = 741. Data from SLOSH

The measurement model showed good fit statistics and there was no indication of measurement variance in the latent variables work-personal life interference and depressive symptoms over time. The structural equation parameters of the bivariate associations between work-personal life interference (WPI) and turnover (TO) are presented separately for men and women in Fig. 1. In both genders there were significant autoregressive paths between work-personal life interference t and work-personal life interference t-1 and among men also between turnover t and turnover t-1. The estimate for the association between work-personal life interference t-1 and turnover t was among men .105 (*p* = .000) and among women .087 (*p* = .001). The paths between turnover t-1 and work-personal life interference t were not significant for any gender. The fit of the gender stratified model was chi-square (132) = 554.148, *p* < .001, RMSEA .042, CFI .977, TLI .968, SRMR .028). The model in which the paths between work-personal life interference and turnover were constrained to be equal between genders showed poorer fit to the data than the model in which these paths were allowed to vary freely between genders. In contrast this was not found for the reverse association.

### Multilevel mediation models for men and women

**Fig. 2:**
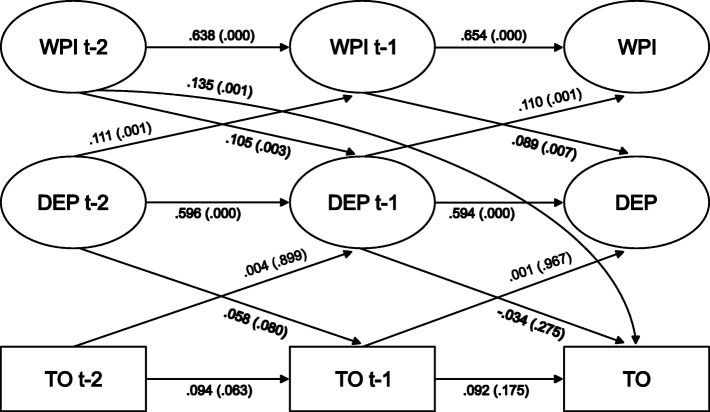
Lags and cross-lags in multilevel structural equation models for paths between work-personal life interference (WPI), depressive symptoms (DEP), and turnover (TO) at time t-2 (ranging from year 2010 to 2012), t-1 (years 2012 to 2014), and t (years 2014 to 2016) among male managers (*n* = 717) in SLOSH

**Fig. 3:**
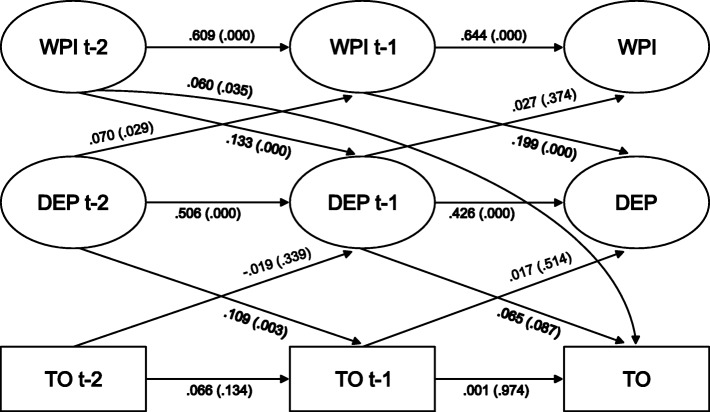
Lags and cross-lags in multilevel structural equation models for paths between work-personal life interference (WPI), depressive symptoms (DEP), and turnover (TO) at time t-2 (ranging from year 2010 to 2012), t-1 (years 2012 to 2014), and t (years 2014 to 2016) among female managers (*n* = 741). Data from SLOSH

The bivariate models estimating the cross-lagged relationships between work-personal life interference and depressive symptoms and between depressive symptoms and turnover showed significant associations for both men and women, suggesting a potential mediating role of depressive symptoms. The model in which the paths between work-personal life interference and depressive symptoms were allowed to vary freely between genders showed significantly better fit to the data than the model in which these paths were constrained to be equal. The same was found for the reverse association. No significant chi square difference was found when comparing constrained and non-constrained models for the paths between depressive symptoms and turnover. The full gender stratified mediation model as well as the direct and indirect effects between work-personal life interference and turnover is presented separately for men (Fig. 2) and women (Fig. 3). All structural equation model parameters with standard errors, 95% confidence intervals and *p* values are shown in Table 2. The fit of the model was chi-square (1186) = 3844.602, *p* < .001, RMSEA .043, CFI .942, TLI .933, SRMR .063. The multiple-group comparison test estimated on the full mediation model showed that the model in which all paths for the hypothesised and reverse associations were constrained to be equal between men and women had significantly worse fit to the data than the model in which these paths were allowed to vary freely (chi-square (1193) 3884.609, *p* < .001, RMSEA .043, CFI .941, TLI .932, SRMR .063, chi square difference 40.007 (7)). The paths were therefore allowed to be freely estimated in the final model.

**Table 2:** Standardized parameters, standard errors, 95% CI and *p*-values among men (*n* = 717) and women (*n* = 741)

	Men			Women		
	B(SE)	95% CI	*p*	B(SE)	95% CI	*p*
*Regression weights – WPI*						
WPI t						
WPI t-1	.654 (.030)	.595; .713	.000	.644 (.025)	.594; .694	.000
Depression t-1	.110 (.032)	.047; .173	.001	.027 (.030)	−.032; .085	.374
Higher age	−.066 (.016)	−.119; −.012	.001	.062 (.025)	−.110; −.013	.013
Not married	.022 (.022)	−.021; .064	.320	−.008 (.021)	−.049; .032	.687
Children living at home	−.001 (.026)	−.053; .051	.971	−.048 (.024)	−.096; .000	.050
Public sector	.011 (.021)	−.030; .052	.601	−.037 (.021)	−.078; .004	.077
Higher education	−.003 (.021)	−.045; .039	.901	.064 (.021)	.023; .104	.002
WPI t-1						
WPI t-2	.638 (.031)	.577; .699	.000	.609 (.032)	.546; .671	.000
Depression t-2	.111 (.034)	.045; .178	.001	.070 (.032)	.007; .133	.029
Higher age	−.043 (.029)	−.100; .014	.136	.003 (.026)	−.048; .055	.902
Not married	.006 (.024)	−.040; .053	.787	−.053 (.024)	−.099; −.006	.026
Children living at home	−.002 (.028)	−.057; .054	.957	−.031 (.027)	−.084; .022	.254
Public sector	.018 (.023)	−.027; .062	.431	−.020 (.024)	−.067; .026	.396
Higher education	.049 (.023)	.004; .095	.034	.057 (.023)	.012; .103	.014
*Regression weights – Depression*						
Depression t						
Depression t-1	.594 (.034)	.527; .661	.000	.426 (.044)	.340; .512	.000
WPI t-1	.089 (.033)	.024; .153	.007	.199 (.035)	.130; .268	.000
Turnover t-1	.001 (.024)	−.045; .047	.967	.017 (.027)	−.035; .070	.514
Higher age	−.082 (.027)	−.135; −.029	.003	−.070 (.026)	−.121; −.018	.008
Not married	.015 (.025)	−.034; .064	.543	.027 (.023)	−.019; .073	.248
Children living at home	−.048 (.026)	−.099; .003	.067	−.002 (.027)	−.055; .051	.937
Public sector	.032 (.025)	−.017; .082	.201	.027 (.024)	−.020; .073	.263
Higher education	−.053 (.023)	−.099; −.008	.021	−.058 (.024)	−.106; −.011	.017
Depression t-1						
Depression t-2	.596 (.042)	.515; .678	.000	.506 (.041)	.425; .586	.000
WPI t-2	.105 (.035)	.036; .173	.003	.133 (.034)	.066; .200	.000
Turnover t-2	.004 (.031)	−.056; .064	.899	−.019 (.020)	−.057; .020	.339
Higher age	−.070 (.027)	−.123; −.017	.009	−.051 (.024)	−.098; −.005	.031
Not married	.013 (.027)	−.040; .065	.639	−.008 (.024)	−.056; .040	.734
Children living at home	−.035 (.024)	−.082; .012	.142	−.022 (.026)	−.073; .030	.416
Public sector	−.011 (.008)	−.028; .005	.182	−.005 (.009)	−.021; .012	.588
Higher education	−.025 (.019)	−.063; .012	.182	−.009 (.017)	−.044; .025	.588
*Regression weights – Turnover*						
Turnover t						
Turnover t-1	.092 (.068)	−.041; .226	.175	.001 (.030)	−.059; .060	.974
Depression t-1	−.034 (.031)	−.094; .027	.275	.065 (.038)	−.010; .141	.087
WPI t-2	. 135 (.038)	.060; .210	.000	.060 (.028)	.004; .116	.035
Higher age	.079 (.028)	.025; .133	.004	.097 (.029)	.040; .154	.001
Not married	−.007 (.027)	−.060; .046	.793	.000 (.027)	−.053; .052	.986
Children living at home	.002 (.028)	−.053; .056	.952	.041 (.029)	−.017; .098	.165
Public sector	−.006 (.010)	−.026; . 015	.587	−.013 (.012)	−.035; .010	.271
Higher education	−.013 (.024)	−.059; .033	.587	−.026 (.024)	−.072; .020	.271
Turnover t-1						
Turnover t-2	.094 (.051)	−.005; .194	.063	.066 (.044)	−.020; .153	.134
Depression t-2	.058 (.033)	−.007; .123	.080	.109 (.036)	.038; .180	.003
Higher age	.010 (.031)	−.050; .070	.742	.051 (.024)	.005; .098	.030
Not married	−.020 (.027)	−.073; .034	.474	.012 (.031)	−.049; .073	.693
Children living at home	−.017 (.044)	−.103; .069	.695	.016 (.034)	−.050; .082	.632
Public sector	−.037 (.028)	−.092; .017	.180	−.006 (.032)	−.067; .056	.861
Higher education	.002 (.033)	−.063; .067	.947	−.010 (.032)	−.073; .053	.754
Indirect effect	.000 (.000)	.000; .000	.617	.000 (.000)	.000; .000	.612

Among men the paths between work-personal interference t-2/t-1 and depressive symptoms t-1/t were significant (.105, *p* = .003/.089, *p* = .007). The inverse associations, between depressive symptoms t-2/t-1 and work-personal interference t-1/t, were also significant (.111, *p* = .001/.110, p = .001). There were no statistically significant paths between depressive symptoms t-2/t-1 and turnover t-1/t or between the variables in the opposite direction. As for men, among women there were statistically significant paths between work-personal life interference at t-2/t-1 and depressive symptoms at t-1/t (.133, *p* = .000; .199, *p* = .000) and a significant path between depressive symptoms t-2 and work-personal life interference t-1 (.070, *p* = .029). The path between depressive symptoms and turnover was significant between t-2 and t-1 (.109, *p* = .003) but not between t-1 and t. There was, as shown in Figs. 2 and 3 and Table 2, a significant direct effect between work-personal interference t-2 and turnover t in both genders (men .135, *p* = .001; women .060, *p* = .035), but as shown in Table 2 no indication of an indirect effect mediated by depressive symptoms in any gender (men *p* = .617, women *p* = .612).

## Discussion

### Summary of main results

In the present study we investigated the longitudinal association between managers’ work-personal interference and turnover, to what extent depressive symptoms mediated this relationship, and if the associations differed by gender. In accordance with our first hypothesis significant prospective paths between work-personal life interference and turnover were found among both male and female managers. There were furthermore, in line with our second hypothesis, significant associations between work-personal life interference and depressive symptoms as well as between depressive symptoms and turnover in bivariate models in both genders. However, no significant intermediate effect of depressive symptoms in the association between work-personal life interference and turnover was found in any gender, and therefore our second hypothesis was not supported. There were gender differences in several of the estimated associations, lending partial support for our third hypothesis.

The turnover rate increased over the study period particularly among female managers. Since we have not investigated this specifically we cannot give any empirically based suggestions as to why this is the case. We can, however, speculate that this may be associated with the fact that the quality of the psychosocial work environment in women-dominated industries, such as education and health care, has decreased continually over the past decades in Sweden [40]. The rate of sickness absences with depression or stress-related diagnoses have increased considerably starting from 2010 in Sweden [41] and the large majority of these absences are in education and health-care, where more female than male managers are employed. This is also the case in the present data. A deteriorating psychosocial work environment and an increasing number of employees on sickness absence due to stress and mental ill-health may have influenced more female than male managers over time to choose to quit their positions.

### Work-personal life interference and turnover

The present study contributes to the current literature by showing that work-personal life interference predicts turnover among both male and female managers in a sample representing a wide range of branches in the whole of Sweden, a result that to the best of our knowledge has not previously been reported. The associations appear to differ between genders; the direct effect between work-personal life interference and turnover was stronger for male than female managers. This may be surprising in the light of previous research suggesting that women are more likely to make changes in their professional career for family reasons [42]. The fact that the work-personal interference scale is focused on an aspired personal life, things one would like to do, and one’s personal needs, rather than family and household obligations, could be an explanation. The results thus support previous research suggesting that women are more likely to neglect personal needs due to work [32] and stay longer in unfavourable work situations [5]. Although it has been reported in previous research on the same study population as the present one that approximately the same proportion of women and men (12–13%) are promoted to a higher position between SLOSH waves, [43, 44] it cannot be ruled out that the women who have chosen to take on a managerial role are a more selected group than the men who have done so. The women may have made more conscious choices, weighing the sacrifices in personal life against the benefits of the position before accepting it, whereas men, due to their lesser engagement in and responsibilities for unpaid household chores, may have been less likely to do so. The more selected group of women who have chosen a managerial career may consequently be more determined to succeed in their role and more reluctant towards abandoning it. It may furthermore to a lesser extent violate male gender role expectations to prioritize their own needs if dissatisfied with their work situation [45]. Among men there was a significant association between turnover t and turnover t-1, indicating that some men change to lower organisational positions repeated times over the study period. In a recently published study on job promotions we found positive associations also of job promotion between waves [44]. These results suggest that the vertical mobility within the workforce is rather high at least in some parts of the labour market.

### Mediation by depressive symptoms

The results from the full mediation model including three time points, in which autoregressive and cross-lagged paths between the variables were estimated, indicate a statistically significant path between work-personal interference and managerial turnover 4 years later among both men and women. This result lends support for that work-personal interference is a relevant long-term predictor of turnover among managers of both genders.

The paths between work-personal interference and depressive symptoms as well as the paths between depressive symptoms and work-personal life interference were significant in both genders. The path from work-personal life interference to depressive symptoms was stronger for female than male managers, a result that is in accordance with previous studies [24, 29]. It has been suggested that female managers are more loyal to their organisations than male managers, particularly to human service organisations where the needs not only of employees but also of recipients of the human services, such as children, pupils, sick, and elderly could be affected [5]. Female manager may, in accordance with what is congruent with a female gender role, [45] to a larger extent than male managers put the needs of those around them at work in the first room and neglect their own needs to the extent where symptoms of depression may develop.

The reverse path, from depressive symptoms to work-personal life interference, was on the other hand stronger among male than female managers. The fact that depressive symptoms affect the perception of stressors has been established previously, [30] and can be one reason for the results. It does not, however, explain the gender differences. Male managers (more often than female managers) with higher levels of depressive symptoms prioritize their work on the expense of their personal life to a higher degree than male managers lower in depressive symptoms. We can only speculate about the reasons. Men higher in depressive symptom scores may be more dissatisfied with their work situation and prioritize work on the expense of personal life in attempts to change their situation. This interpretation would be in line with research showing that men in lower positions show more depressive symptoms than men in higher positions, a result seldom seen among women [18, 46].

In the bivariate models the association between depressive symptoms and turnover was significant in both genders, supporting previous research indicating mental ill-health as important contributors to turnover [9, 27]. However, although significant paths were found between work-personal life interference and depressive symptoms and between depressive symptoms and turnover in the bivariate analyses, no significant mediation through depressive symptoms was observed in the full models for any gender. Thus, symptoms of depression were not found to be an important mechanism in the association between work-personal interference and actual turnover. However, there are indications in line with our second hypothesis, that depressive symptoms play a role in this association, and furthermore the third hypothesis that depressive symptoms play a somewhat stronger role for female managers in the turnover process. Depressive symptoms were significantly associated with turnover between two waves among women, but not among men, and additionally the direct path between work-personal interference and turnover 4 years later was somewhat stronger among men than among women. These results are in line with previous reflections suggesting that female managers may be a more selected and dedicated group, often working in human service organisations where the needs of others are quite visible, and in congruence with gender role expectations may prioritise others to the extent where symptoms of depression develop. Whereas increased mental ill-health to a larger extent may force female managers to leave their managerial careers, the process appears to be different for male managers. Male managers may not treasure a managerial position to the same extent as female managers and may choose to leave their position because it interferes with their personal life aspirations. To our knowledge, the only study with a similar focus as the present one, is one on leaders in U.S. addiction treatment organizations presented by Knudsen et al. [9]. This study showed that emotional exhaustion partially mediated the association between job demands and turnover intentions. There are, however, several differences between their study and ours that make them less comparable. Apart from focusing different predictors we measured actual turnover as opposed to turnover intentions, and the two constructs have been shown to measure distinctly different phenomena [5].

### Strengths and limitations

One strength of the present study is that it is one of the first to investigate the association between work-personal life interference and actual managerial turnover under the consideration of mediation by depressive symptoms. Further strengths are the longitudinal design, the data comprising managers from a wide range of branches from the whole of Sweden, and the measure of actual turnover. A limitation is that we have not been able to distinguish between voluntary and involuntary turnover. Also, we do not know if two years, which is the lag between waves in the SLOSH study, is the optimal time frame to capture the development of associations between experienced work-personal life interference, depressive symptoms, and changed job position. In the present study we used advanced statistical mediation methodology, which combine multilevel and SEM models and thereby allow correction for sampling and measurement error, as well as examination of direct and indirect effects at each level [38]. We included measurements at three waves as suggested in mediation modelling literature in order to appropriately incorporate the temporal ordering between the three measures [47]. Finally, we took into account bidirectional associations allowing correlations between all constructs and the errors of individual items over time to account for consistency in item-specific variance, which improve our understanding of cause and effect [34, 48].

## Conclusions

Establishing organisational prerequisites for good work-personal life balance among managers may be a means to retain both male and female managerial talent.

## Abbreviations

ANOVA: One-way analysis of variance
CFI: The comparative fit index
DEP: Depressive symptoms
MSEM: Multilevel structural equation modelling
RMSEA: The root mean square error of approximation
SCL-CD_6_: Symptom Checklist Core Depression Scale
SLOSH: Swedish Longitudinal Occupational Survey of Health
SRMR: The standardized root mean square residual
SWES: Swedish Work Environment Surveys
TLI: The Tucker-Lewis index
TO: Turnover
WPI: Work-personal life interference
